# The Effects of Aluminum-Nitride Nano-Fillers on the Mechanical, Electrical, and Thermal Properties of High Temperature Vulcanized Silicon Rubber for High-Voltage Outdoor Insulator Applications

**DOI:** 10.3390/ma12213562

**Published:** 2019-10-30

**Authors:** Chang Liu, Yiwen Xu, Daoguang Bi, Bing Luo, Fuzeng Zhang, Tingting Wang, Yingbang Yao, Shengguo Lu, Wenrong Xu

**Affiliations:** 1 School of Electric Power, South China University of Technology, Guangzhou 510641, China; liuchang@csg.cn; 2 China Southern Power Grid, Guangzhou 510623, China; luobing@csg.cn (B.L.); zhangfz@csg.cn (F.Z.); wangtingting@csg.cn (T.W.); 3 School of Materials and Energy, Guangdong University of Technology, Guangzhou 510006, Chinam13113987120@163.com (D.B.); sglu@gdut.edu.cn (S.L.); 4 CYG Insulator Co. Ltd., Dongguang 523128, China; xuwenrong@cyginsulator.com

**Keywords:** AlN nanoparticle, silicon rubber composite, high-voltage outdoor insulators

## Abstract

AlN nanoparticles were added into commercial high-temperature-vulcanized silicon rubber composites, which were designed for high-voltage outdoor insulator applications. The composites were systematically studied with respect to their mechanical, electrical, and thermal properties. The thermal conductivity was found to increase greatly (>100%) even at low fractions of the AlN fillers. The electrical breakdown strength of the composites was not considerably affected by the AlN filler, while the dielectric constants and dielectric loss were found to be increased with AlN filler ratios. At higher doping levels above 5 wt% (~2.5 vol%), electrical tracking performance was improved. The AlN filler increased the tensile strength as well as the hardness of the composites, and decreased their flexibility. The hydrophobic properties of the composites were also studied through the measurements of temperature-dependent contact angle. It was shown that at a doping level of 1 wt%, a maximum contact angle was observed around 108°. Theoretical models were used to explain and understand the measurement results. Our results show that the AlN nanofillers are helpful in improving the overall performances of silicon rubber composite insulators.

## 1. Introduction

Nowadays, more and more composite insulators are used for the supporting parts of outdoor high-voltage transmission lines and equipment, due to their unique virtues, such as being light-weight, having a high electrical breakdown strength, hydrophobic nature, good flexibility, and easy processing, compared to the conventional glass or ceramic insulators [1]. Silicon rubber is the most popular polymer matrix for this application [1,2]. Aluminum hydroxide and silicon dioxide are usually added into silicone rubber to introduce a flame-retardant property and increase the tracking and erosion resistance of the composite insulators [3]. Meanwhile, the thermal conductivity will also be increased due to the high thermal conductivity inorganic fillers [4]. It was shown that thermal accumulation is one major cause for the accidental failures of outdoor composite insulators [5], and silicon rubber has a very low thermal conductivity (~0.3 W/m·K), which is not in favor of thermal dissipation. Therefore, composite insulators with high thermal conductivity are desired in order to reduce those accidental failures. Inorganic fillers with much higher thermal conductivity are usually added into the polymer to increase the thermal conductivity of the composites [6]. 

Previous studies have revealed that the surface area and shape of the fillers are two important factors affecting the thermal conductivity of the final composite materials [7]. The interface between the inorganic filler and the polymer matrix also plays an important role in determining the thermal, electrical, mechanical, and other physical properties of the composite [8]. Large-size fillers (>1 μm) are usually better for thermal conductivity improvement because of their lower interfacial area, which usually behaves as a resistant barrier for thermal conduction [9]. However, large particles will deteriorate the mechanical properties of the composites and tend to introduce more voids in the composite [10,11]. On the other hand, nanofillers will enhance the thermal conductivity considerably at a much lower volume ratio due to the easily formed thermal conduction path, i.e., percolation behavior [12]. In some cases, it was shown that using a mixture of fillers with different sizes, i.e., nano and micro, will lead to a better thermal performance as compared to using only one [13].

For high-voltage outdoor composite insulators, a high thermal conductivity and good electrical insulation are required. Under such limits, there are not too many choices for ceramic fillers. BN (thermal conductivity of 29–300 W/m·K), Al_2_O_3_ (38–42 W/m·K), AlN (150–220 W/m·K), BeO (300 W/m·K), SiC (85 W/m·K), and Si_3_N_4_ (86–120 W/m·K) have attracted much attention with respect to such applications [5,6,14]. Among these candidates, BeO and BN exhibit the best thermal conductivity and also well-known electrical insulation properties. However, BeO is toxic and BN is expensive. In the current report, AlN was chosen as the ceramic filler due to its high thermal conductivity, good electrical insulation, and economic cost. 

Namitha et al. studied the effects of micrometer-sized AlN particle (<50 μm) fillers on the dielectric properties in the microwave frequency range and thermal properties of silicon rubber composites [15]. They obtained a thermal conductivity of ~1.2 W/m·K at a filling ratio of 41 vol%. Chiu et al. coated the micron AlN with SiOC or SiONC film to increase the compatibility with the silicon rubber matrix and an enhancement of the thermal conductivity (0.89 W/m·K at filler fraction of 30 vol%) was observed [16]. Nazir et al. added micron AlN and nano SiO_2_ simultaneously into silicon rubber and studied the dielectric properties of the composites [11]. The co-filled samples exhibited better thermal stability than those samples filled with only one kind of filler, i.e., either micron AlN or nano SiO2, but no thermal conductivity data were given. They also found that with increasing the micron AlN content (from 5 wt% to 30 wt%), the high-voltage electrical tracking behavior was exacerbated [17]. Tian et al. modified the surface of micron AlN particles with KH550 coupling agent and obtained a thermal conductivity of 1.3 W/m·K at a filling level of 28.5 wt% in silicon rubber [10].

Most of the above-mentioned studies on AlN-polymer composites focused on their applications as thermal interface materials (TIM), which is usually in the thermal management area of integrated circuits (IC) [18]. It is obvious that AlN-filled silicon rubber has not been systematically investigated with respect to its application as a high-voltage outdoor insulator. In such applications, thermal, mechanical, electrical, dielectric, and hydroph obic properties should be systematically studied. Moreover, nanometer-sized AlN has rarely been used as the fillers and it deserves a thorough study. Therefore, in the current report, we used nano AlN as the filler, and prepared a series of samples with different filling ratios (0 wt%, 1 wt%, 2 wt%, 3 wt%, 4 wt%, 5 wt%, 6 wt%, 8 wt%, and 10 wt%, corresponding to 0 vol%, 0.5 vol%, 1.0 vol%, 1.5 vol%, 2.0 vol%, 2.5 vol%, 3.0 vol%, 4.0 vol%, and 5.1 vol%, respectively). Since at higher filling ratios, the flexibility of the composites will degrade seriously, we focused on lower filling ratios in the current work, i.e., below 5 vol%. Their thermal, mechanical, electrical, dielectric, and hydrophobic properties were tested. We found that AlN nanofillers are very effective in enhancing the thermal conductivities of silicon rubber composites, which will benefit their applications as outdoor high-voltage insulators.

## 2. Materials and Methods 

AlN nanoparticles were purchased from Shanghai CWNano Ltd. The nominal averaged diameter was 50 nm, but with severe agglomeration, as will be shown later. The composite silicone rubber samples were prepared by CYG Insulators Co., Ltd. in Dongguan, Guangdong, China. Firstly, the AlN nanoparticle fillers were mechanically mixed with other raw materials (methyl vinyl silicone rubber, SiO_2_, Al(OH)_3_, hardener additive) in a kneading machine and then a three roll milling machine (repeated eight times) to get a homogeneous dough, and then underwent high temperature vulcanization (10 MPa, 180 °C, 60 min). The AlN filler fraction varied from 0 wt% (sample #1), 1 wt% (#2), 2 wt% (#3), 3 wt% (#4), 4 wt% (#5), 5 wt% (#6), 6 wt% (#7), 8 wt% (#8), and 10 wt% (#9). The corresponding volume ratios were calculated to be 0 vol% (#1), 0.5 vol% (#2), 1.0 vol% (#3), 1.5 vol% (#4), 2.0 vol% (#5), 2.5 vol% (#6), 3.0 vol% (#7), 4.0 vol% (#8), and 5.1 vol% (#9), respectively.

The densities of the samples were measured by Archimedes method. The mechanical properties, such as Shore hardness, tensile strength, tear strength, tension set, and elongation at break, were measured according to the national standards of China GB/T528-2009 and GB/T33429-2016. These standards are compatible with the international standards of ISO 37:2005, ISO 34-1:2004, ISO 2285:2007, and ASTM D2240-2015. Five samples for each composition were tested for every single measurement. The electrical breakdown strength was measured, complying with the national standards of China GB/T19519-2004. The electrical field was applied at a constant rate of 1 kV/s. Five samples for each composition were tested for one measurement. The electrical tracking and erosion performance was tested according to the national standards of China GB/T6553-2014. Five samples for each composition were measured. The hydrophobicity was evaluated by measuring the temperature-dependent contact angles for all the samples (Dataphysics OCA-50). The thermal properties, heat capacity and thermal diffusivity, were measured by the differential scanning calorimetry (DSC) method (NETZSCH DSC-214) and laser flash spectrometer (NETZSCH LFA-447), respectively. The thermal conductivity was calculated by: κ = αρCp, where κ, α, ρ, and Cp are the thermal conductivity (W/m·K), thermal diffusivity (mm^2^/s), density (g/mm^3^), and heat capacity (J/g·K), respectively. The microstructures of the samples were examined by scanning electron microscopy and transmission electron microscopy (SEM: JSM-7500, TEM: JEM-2100F, JEOL, Tokyo, Japan). The dielectric properties were measured by an LCR (L: Inductance, C: Capacitance, R: Resistance) meter (Hioki IM3536, Hioki, Nagano, Japan).

## 3. Results and Discussions

### 3.1. Microstructures

**Figure 1:**
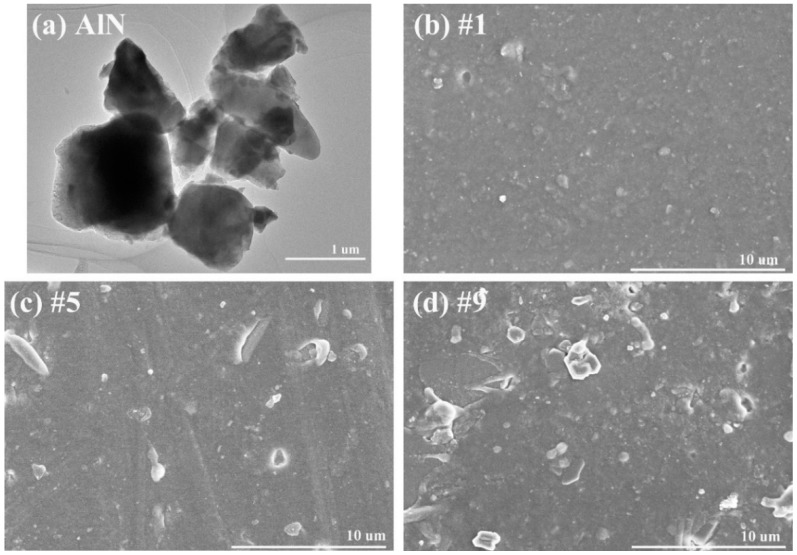
TEM micrograph of (**a**) as-received AlN nanoparticles, and SEM micrographs of sample (**b**) #1, (**c**) #5, and (**d**) #9.

The TEM micrograph of the as-received AlN nanoparticles is shown in Figure 1a. One can find that the size of the AlN particles was not uniform. Small nanoparticles (50–500 nm) were agglomerated together and there were also some bigger particles with diameters of 1–2 μm, which is much larger than the nominal size (50 nm). The SEM micrograph of the pure silicone rubber sample (#1) is shown in Figure 1b. There are some particles on the surface, which are believed to be the silicon dioxide and aluminum hydroxides, as per the receipt. With increasing the AlN filler’s volume fraction, the surface of the sample became more rough, as shown in Figure 1c,d for sample #5 and sample #9. Moreover, there appears some voids between the filler and the matrix. This may be partially due to the absence of coupling agent on the surface of the AlN particles. We used the AlN particles as received without any further treatment. Such results indicate that the coupling between the AlN filler and the silicone rubber matrix was very weak. In future, we need to modify the surface of the AlN particles in order to obtain more dense and compact microstructures. 

**Figure 2:**
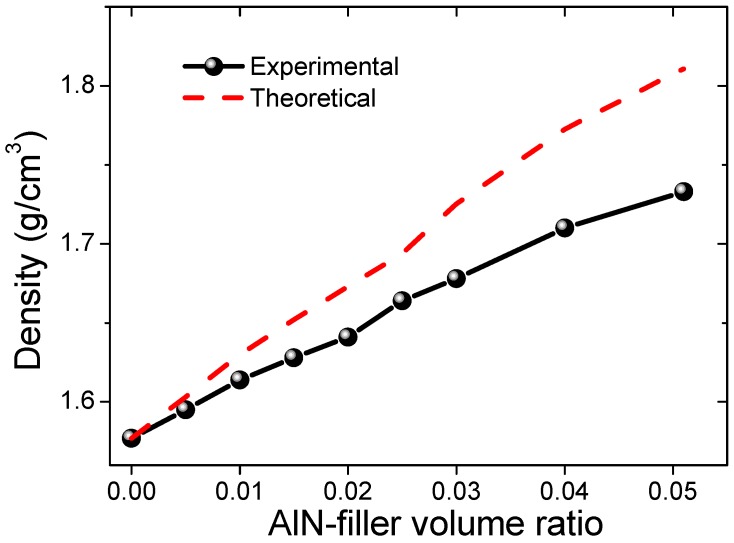
Density of the AlN-filled silicon rubber composite samples.

The densities of all the samples are shown in Figure 2. The theoretical values were calculated by the following equation:

(1) $$\rho =\left(1-V_{f}\right)\cdot \rho_{m}+V_{f}\cdot \rho_{f},$$

where *ρ*, *ρ*_m_, *ρ_f_* are the density of the composite, polymer matrix, and the filler, respectively, and *V_f_* is the volume ratio of the filler [19]. Using the densities of current silicone rubber sample (1.57 g/cm^3^) and the pure AlN (3.26 g/cm^3^), the theoretical densities of all samples were thus obtained and also shown in Figure 2. The profile of the measured densities is consistent with the theoretical calculations. However, it is obvious that the measured density was lower than the theoretical one. Moreover, with increasing the filler’s ratio, the difference between the measured density and the theoretical density became larger. This is consistent with the SEM observations. With increasing the AlN volume ratio, more voids were formed, as shown in Figure 1, thus deviating more from the theoretical predictions.

### 3.2. Mechanical Properties

**Figure 3:**
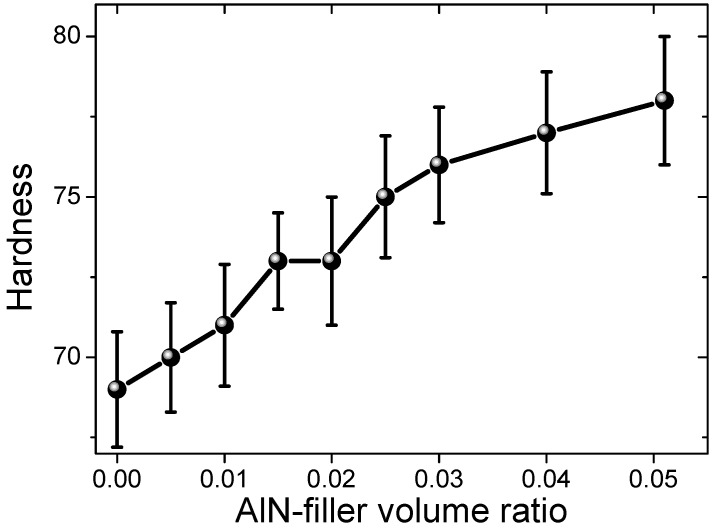
Shore hardness (HA) of the AlN-filled silicon rubber composite samples.

The Shore hardness (HA) of all the samples is shown in Figure 3. The hardness of the unfilled silicon rubber was 69. With increasing the filler’s ratio, the hardness was gradually increased to 78 for sample #9, as shown in Figure 3. The increase in hardness with the filling ratio of the inorganic fillers is normal and has been observed in many other composites [5]. This is due to the fact that inorganic ceramic fillers are usually harder than the polymer matrix.

**Figure 4:**
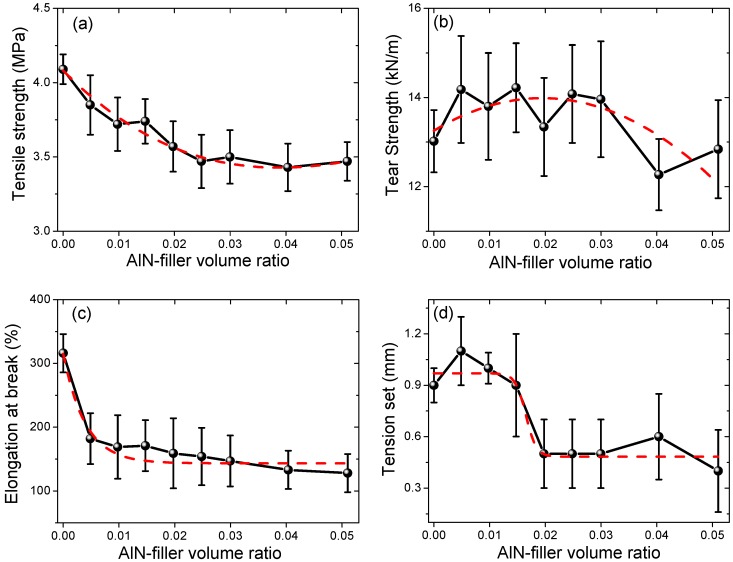
(**a**) Tensile strength, (**b**) tear strength, (**c**) elongation at break, and (**d**) Tension set of the AlN-filled silicon rubber composite samples. The dashed lines in the figures are for eye-guidance.

The tensile strength, tear strength, elongation at break, and tension set of all the samples are shown in Figure 4. The tensile strength of the virgin silicon rubber sample #1 was 4.09 MPa and with the AlN filler’s fraction increasing, the tensile strength decreased gradually to 3.37 MPa for sample #6 (filler ratio of 2.5 vol%). Further increasing the AlN filler ratio did not change the tensile strength much, as shown in Figure 4a. The tear strength of the unfilled sample #1 was 13.02 kN/m, which then increased gradually with the AlN filler’s ratio, reaching a maximum value of 14.68 kN/m for sample #6. After that, the tear strength decreased with the filling ratio, as shown in Figure 4b. For example, the tear strength was decreased to 12.84 kN/m for sample #9. The elongation at break for the unfilled silicon rubber sample #1 was 316%, which is a normal value [5]. However, after adding the AlN filler, the elongation at break decreased abruptly to only 182% for sample #2 (0.5 vol%). Then, it decreased gradually to 128% for sample #9 (5.1 vol%), as shown in Figure 4c. This means that the AlN filler deteriorates the flexibility of the silicon rubber composites significantly, even at a filling ratio as low as 0.5 vol%. The tension set of sample #1 was 0.9 mm. After adding the AlN filler, it did not change much until the filling ratio of 1.5 vol% (sample #4). Then, it decreased abruptly to 0.5 mm for sample #5 (2.0 vol%) and stayed almost constant with further increasing of the filling ratio, as shown in Figure 4d.

**Figure 5:**
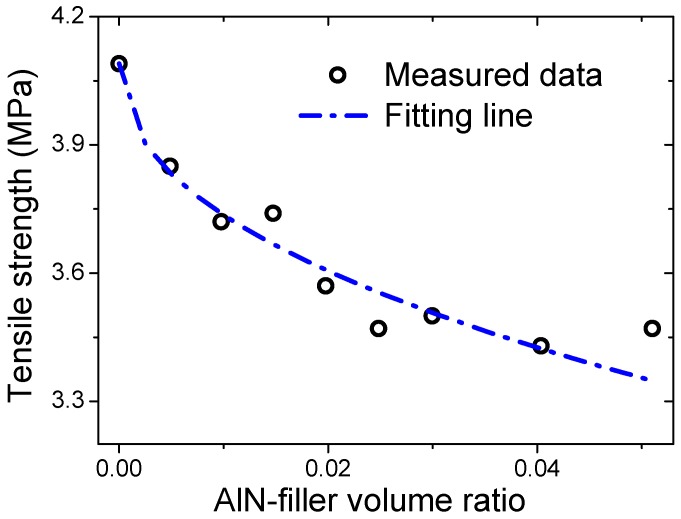
Fitting results of the tensile strength of the composites based on a power-law model.

The tensile strength of composites can be described by a power-law model,

(2) $$\sigma_{c}=\sigma_{m}\left(1-a\cdot V_{p}^{b}\right),$$

where *σ_c_*, *σ_m_*, *V_p_* are the strength of the composite, the strength of polymer matrix, and volume filling ratio of the filler, respectively, and *a* and *b* are constants [20]. The fitting results are shown in Figure 5. The fitting constants were determined to be 0.70 and 0.45 for *a* and *b*, respectively, which are comparable with those for BN-filled silicon rubber samples [5]. The determination of coefficient of the fitting was 0.81. It was shown that the fitting curve coincided reasonably well with the measurement data. In order to gain further insight into the effects of the AlN fillers on the mechanical properties of the silicon rubber composites, more detailed studies will be needed. For instance, the voids between the AlN particles and the polymer matrix, the distribution of the AlN particles, and other defects should be worthy of much attention.

### 3.3. Electrical Properties

**Figure 6:**
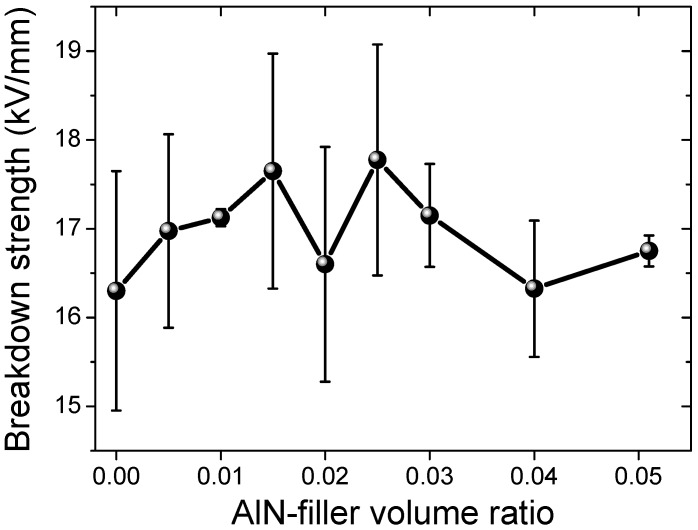
Electrical breakdown strength of the AlN-filled silicon rubber samples.

Electrical breakdown strengths of all the samples are shown in Figure 6. With the addition of AlN, the electrical breakdown strength first increased, i.e., from 16.3 kV/mm for sample #1 to 17.6 kV/mm for sample #4 (1.5 vol%), and then decreased gradually to 16.7 kV/mm for sample #9 (5.1 vol%). The increase of the electrical breakdown strength with the filling ratios (when the filling ratio is low) was also observed previously in other inorganic-polymer composites, which was attributed to the trapping of the mobile electrical charge carriers at the interface of the ceramic filler and the polymer matrix [5,21,22,23]. After filling with the inorganic particles, the thermal conduction will also be increased and better heat dissipation can also enhance the electrical breakdown strength [24], while at high filling ratios, the ceramic particles will agglomerate and the distance between them will be reduced, thus facilitating the electrical charge transportation, i.e., deteriorating the insulating properties. Moreover, at higher loading ratios, more voids will be introduced into the samples, which will introduce more defects, leading to a lower electrical breakdown strength. 

**Figure 7:**
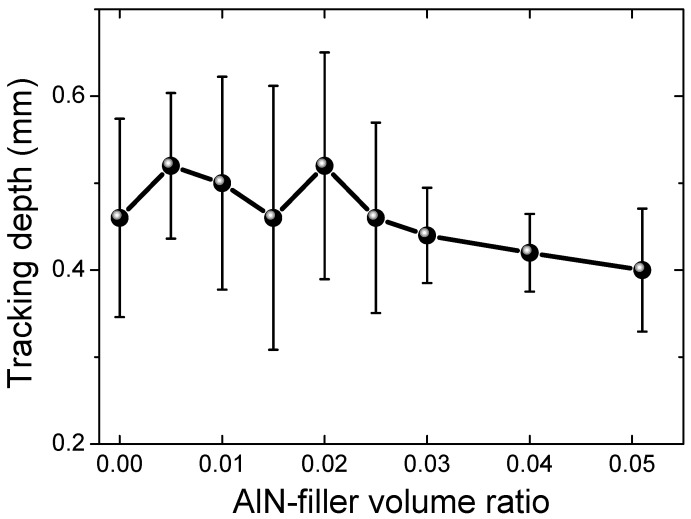
Electrical tracking and erosion measurement results (depth in mm) of the AlN-filled silicon rubber samples.

The electrical tracking and erosion measurement results are shown in Figure 7. The measurement voltage was set to 4.5 kV. The unfilled silicon rubber sample #1 exhibited an erosion depth of 0.46 mm. With increasing the AlN filler’s fraction, the erosion depth was increased to 0.52 mm for sample #5 (2.0 vol%). Further increasing the AlN filling ratio led to a gradual decrease of the erosion depth, finally reaching 0.40 mm for sample #9, as shown in Figure 7. The defects in the samples, induced by the addition of AlN filler, would exacerbate the electrical tracking and erosion, i.e., the increase in the erosion depth with the filling ratio from 0 vol% (sample #1) to 2.0 vol% (sample #5). Although at higher filling ratios more defects will be introduced in the composite samples, the thermal conduction will also be increased (to be shown later) and can mitigate the erosion through fast local heat dissipation. Therefore, the decrease in the erosion depth with the increase in the filling ratios, i.e., from 2.0 vol% (sample #5) to 5.1 vol% (sample #9), may be mainly related to the increase in the thermal conduction of the samples.

### 3.4. Hydrophobic Properties

**Figure 8:**
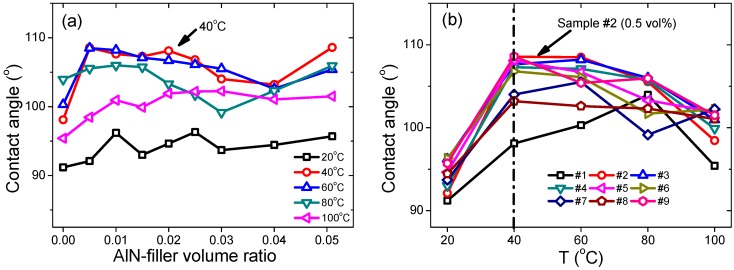
(**a**) Filler-ratio-dependent contact angles at different temperatures, and (**b**) temperature-dependent contact angels of each sample. The dashed lines indicate the sample or temperature where the maximum contact angle happened.

The hydrophobic properties of our composite samples were studied through the measurement of their contact angles. Temperature-dependent contact angles of all the samples are shown in Figure 8. At 20 °C, sample #1 showed a contact angle of 91.2°, which is compatible with previously published results [5]. With increasing the AlN filling ratio, the contact angle was increased to 96.3° for sample #6 (2.5 vol%), and then decreased to 95.7° for sample #9 (5.1 vol%), as shown in Figure 8a. As the measurement temperature increased to 40 °C, a maximum contact angle of 108.5° was observed in sample #2. With the temperature increased further, the contact angle was found to be decreased for all samples except that of the sample #1. And in the whole range of temperatures, sample #2/#3 exhibited the maximum value of contact angle, which proves that samples #2 and #3 had the best hydrophobic properties. The contact angle of sample #1 also increased with the measurement temperature, and reached the maximum value of 104.0° at 80 °C, and then decreased to 95.4° at 100 °C. The temperature, where the maximum contact angle was observed, was found to be 40 °C for samples #2, #4, #5, #6, #8, and #9, as shown in Figure 8b. For samples #3 and #7, the maximum contact angle was observed at the temperature of 60 °C. Thus, one can say that with the AlN filler, the hydrophobic properties were obviously improved in a wide temperature range and the behavior of the hydrophobic property with temperature was also changed, i.e., the temperature of maximum contact angle shifted from 80 °C for sample #1 to lower temperatures of 40–60 °C for other samples. The origin of such changes was yet not clear in the current stage and deserves more detailed studies in future. Nonetheless, our results may provide some useful hints for practical applications.

### 3.5. Thermal Properties

**Figure 9:**
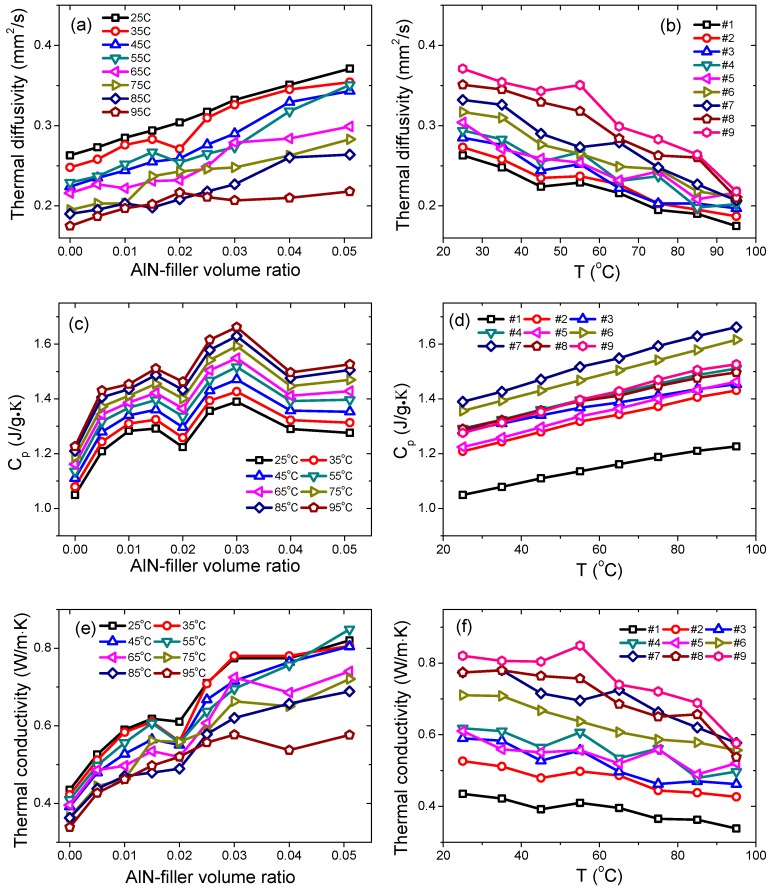
Temperature-dependent (**a**,**b**) thermal diffusivity, (**c**,**d**) heat capacity, and (**e**,**f**) thermal conductivity of the AlN-filled silicon rubber composites.

The temperature-dependent thermal diffusivity results of all the samples are shown in Figure 9a,b. The thermal diffusivity of sample #1 at 25 °C was 0.26 mm^2^/s, and after adding AlN fillers, the thermal diffusivity was increased to 0.37 mm^2^/s for sample #9 (5.1 vol%), as shown in Figure 9a. At higher temperatures, similar behavior was observed, i.e., larger thermal diffusivity at higher AlN filling ratios. For a specific sample, the thermal diffusivity decreased with the temperature, as shown in Figure 9b. For instance, for sample #1, the thermal diffusivity was decreased from 0.26 mm^2^/s at 25 °C to 0.17 mm^2^/s at 95 °C (difference ~34%), and for sample #9, from 0.37 mm^2^/s at 25 °C to 0.22 mm^2^/s at 95 °C (difference ~41%). The changing rate, or slope, of thermal diffusivity versus temperature was increased with the AlN filling ratio, as observed in Figure 9b.

The specific heat capacities (C_p_) of all samples are shown in Figure 9c,d. With increasing the AlN filler’s fraction, the heat capacity was found to be increased from 1.05 J/g·K for sample #1 (25 °C) to 1.39 J/g·K for sample #7 (3.0 vol%), and then decreased gradually with the filler’s fraction, finally reaching 1.27 J/g·K for sample #9 (5.1 vol%). At other temperatures, i.e., from 25 °C to 95 °C, a similar trend was evidenced, as shown in Figure 9c. An almost linear relationship for heat capacity versus temperature was observed for all samples in the temperature range of from 25 °C to 95 °C. For example, the C_p_ of sample #1 was increased from 1.05 J/g·K at 25 °C to 1.23 J/g·K at 95 °C (change ~17%), and C_p_ of sample #9 was increased from 1.27 J/g·K at 25 °C to 1.52 J/g·K at 95 °C (change ~19%). The changing rate of the C_p_ with temperature was almost the same for all the samples, as shown in Figure 9d. 

**Figure 10:**
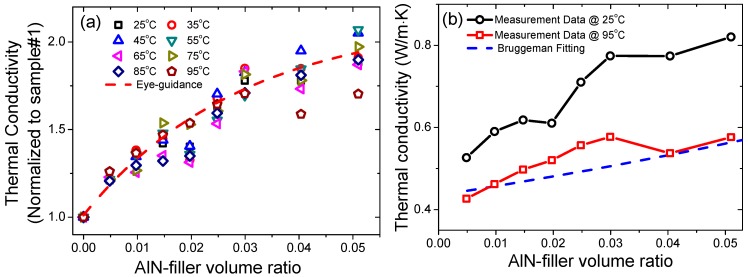
(**a**) Thermal conductivity data normalized to that of the sample #1, (**b**) Bruggeman fitting for the thermal conductivity data.

The thermal conductivity data are shown in Figure 9e,f. At 25 °C, the thermal conductivity of sample #1 was 0.43 W/m·K, which was increased gradually with the filler’s fraction to 0.82 W/m·K for sample #9 (5.1 vol%), as shown in Figure 9e. Similar behavior was observed at other temperatures (up to 95 °C). For sample #1, the thermal conductivity was decreased from 0.43 W/m·K at 25 °C to 0.34 W/m·K at 95 °C (difference ~23%). On the other hand, for sample #9 (5.1 vol%), the thermal conductivity was decreased from 0.82 W/m·K at 25 °C to 0.57 W/m·K at 95 °C (difference ~ 30%), as shown in Figure 9f. In order to emphasize the effects of the AlN filler on the thermal conductivity of the silicon rubber composites, the data were normalized with respect to the thermal conductivity of the sample #1 and the data are re-plotted in Figure 10a. It is obvious that the AlN filler was very efficient in enhancing the thermal conductivity of the silicon rubber composites, even at very low filling ratios. At 25 °C, a maximum increment of 88% was obtained in sample #9 (5.1 vol%). Moreover, even at a low filling ratio of 1.5 vol% (sample #4), the enhancement was still as large as 42%, as shown in Figure 10a. Moreover, at measurement temperatures of 45 °C and 55 °C, the enhancement of the thermal conductivity was more than 100%, i.e., 105% and 107%, respectively, as shown in Figure 10a. Our results are comparable to the published results related to the AlN-filled polymer composites, such as AlN-epoxy and AlN-silicone rubber [7,8,10,15,16], but with a much lower volume ratio of the AlN filler in our study. Such differences may come from the smaller dimensions of the AlN particles in current case, i.e., nano versus micro.

For ceramic-polymer composites, there are many theoretical models for the understanding of the thermal conductivity behavior [25,26,27,28]. Among them, the Bruggeman model is the most popular. In the Bruggeman model, the thermal conductivities of the composite, particle filler, and polymer matrix are related by the following equation [26]:

(3) $$1-\emptyset =\frac{k_{p}-k_{c}}{k_{p}-k_{m}}\cdot {\left(\frac{k_{m}}{k_{c}}\right)}^{1/3},$$

where *k_p_*, *k_m_*, *k_c_*, and ∅ are thermal conductivity of the particle filler (200 W/m·K for current case), polymer matrix (0.43 W/m·K for current case), composite, volume ratio of the particle filler, respectively. The fitting results are illustrated in Figure 10b. One can find that at room temperature (25 °C), the measurement data were much larger than those of theoretical calculations. However, at higher temperatures, i.e., 95 °C, the measurement data matched reasonably well with the theoretical calculations, as shown in Figure 10b. The voids in our samples and other additives during the processing (the quantity of those fillers in the proprietary recipe was not disclosed) may account for such deviations, because they will directly affect the value of the volume ratios in the theoretical model. Thus, the theoretical model also needs to be modified in order to account for the effects of more than one filler, e.g., voids, AlN nanoparticles, other kind of filler particles. We will endeavor to investigate such theoretical work in the next step.

### 3.6. Dielectric Properties

**Figure 11:**
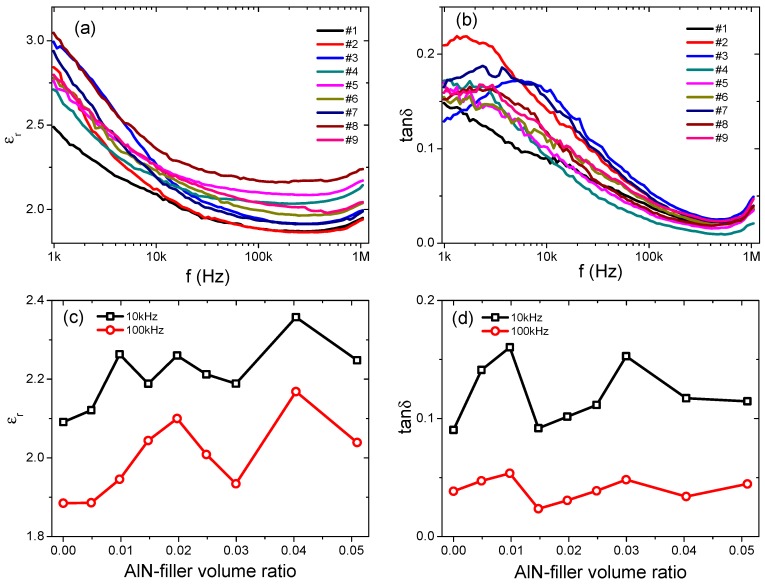
Frequency-dependent of (**a**) dielectric constants, and (**b**) dielectric losses of the AlN-filled silicon rubber composites. Filling-ratio-dependent of (**c**) dielectric constants, and (**d**) dielectric losses at 10 kHz and 100 kHz.

The frequency-dependent dielectric constants (*ε_r_*) and losses (tan δ) of all the samples are shown in Figure 11a,b. For all samples, the dielectric constants decreased with frequency. For sample #1, ε_r_ was decreased gradually from 2.49 at 1 kHz to 1.94 at 1 MHz, while tanδ was decreased from 0.148 at 1 kHz to 0.036 at 1 MHz. With increasing the AlN content, the dielectric constant was generally enhanced, as summarized in Figure 11c,d for the filling-ratio-dependent dielectric constant/loss. Among all these samples, sample #8 (4.0 vol%) exhibited the largest *ε_r_* of 2.36 at 10 kHz and 2.17 at 100 kHz. For sample #9 (5.1 vol%), the dielectric constant was decreased to 2.25 at 10 kHz and 2.04 at 100 kHz, which may be related to the greater number of voids introduced into the sample under high AlN filling ratios. Generally, with increasing the AlN filler fraction, the dielectric loss was increased, as shown in Figure 11b,d. The tanδ was 0.090 and 0.038 for sample #1 at 10 kHz and 100 kHz, respectively. For sample #8, tanδ was 0.117 and 0.034 at 10 kHz and 100 kHz, respectively. Moreover, relaxation peaks in the frequency-dependent loss were clearly observed in sample #2 and sample #3 in the current range of measurement frequency (1 kHz–1 MHz). This may be related to the interfaces between the AlN fillers and the silicon rubber matrix, where defect dipoles are usually present [5].

**Figure 12:**
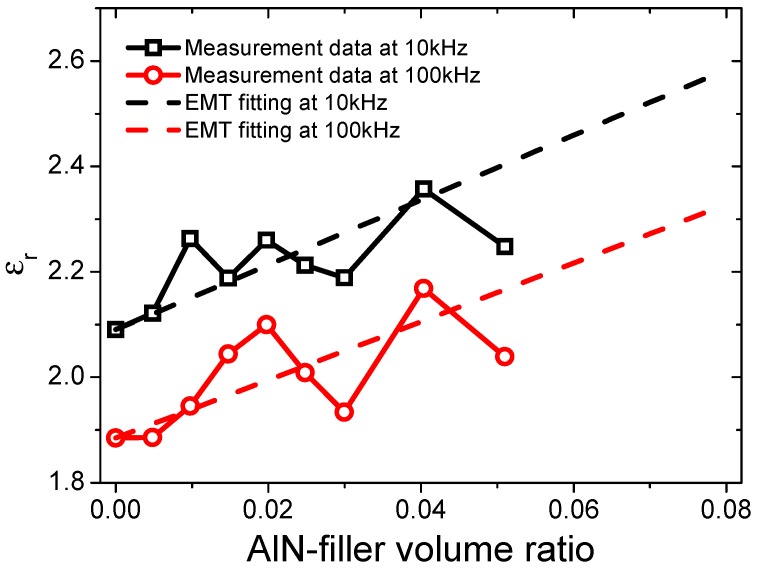
Fitting results for the dielectric constants of the AlN-filled silicon rubber composites based on the effective medium theory.

For ceramic–polymer composites, the effective medium theory is usually used to calculate the dielectric constant of the composite samples [19,29]. Based on this theory, the dielectric constant of the composite can be written as:

(4) $$\varepsilon_{c}=\varepsilon_{m}\left[1+\frac{\emptyset \left(\varepsilon_{p}-\varepsilon_{m}\right)}{\varepsilon_{m}+m\left(1-\emptyset \right)\left(\varepsilon_{p}-\varepsilon_{m}\right)}\right],$$

where *ε_c_*, *ε_m_*, and *ε_p_* are the relative dielectric constants of the composite, polymer matrix (2.09 at 10 kHz and 1.88 at 100 kHz), and particle filler (8.8 for AlN), respectively, and ∅ is the filling fraction in volume. The fitting results are shown in Figure 12. One can find that the theoretical model can fit the experimental data reasonably well with a fitting parameter, *m*, of 0.04. In the effective medium theory, *m* is a morphology factor. The small value of *m*, i.e., 0.04 for our fitting, means the filler particles are close to a spherical shape [19,30], which is consistent with our microstructural observations.

## 4. Conclusions

AlN nanoparticles were added into commercial silicone rubber composites used for outdoor high-voltage insulators. Different filling ratios (0–10 wt% or 0–5.1 vol%) were prepared and tested in order to investigate their effects on the performance of the silicone rubber composites. Their microstructural, mechanical, thermal, hydrophobic, electrical, and dielectric properties were systematically studied. It was found that the mechanical strength and flexibility was decreased by the AlN filler. However, the thermal conductivity was obviously enhanced, i.e., up to 100% increment with respect to that of the unfilled sample. Moreover, the hydrophobic properties were also improved with the increased contact angle after adding the AlN filler. The dielectric constants and losses were increased in the samples with AlN filling. Based on the measurement results, different theoretical models were tested with respect to the tensile strength, thermal conductivity, and dielectric constants. It was verified that the power-law model can fit the tensile strength data well. Our results prove that AlN nanoparticles are very effective in improving the thermal and hydrophobic properties of silicone rubber composites, even at a very low filling ratio, which will be helpful for high-voltage outdoor insulators.
